# Coexistence of sexual individuals and genetically isolated asexual counterparts in a thrips

**DOI:** 10.1038/srep03286

**Published:** 2013-11-21

**Authors:** Kazuya Kobayashi, Jin Yoshimura, Eisuke Hasegawa

**Affiliations:** 1 Laboratory of Animal Ecology, Department of Ecology and Systematics, Graduate School of Agriculture, Hokkaido University, Sapporo, Hokkaido 060-8589, Japan; 2 Department of Mathematical and Systems Engineering, Shizuoka University, Hamamatsu 432-8561, Japan; 3 Marine Biosystems Research Center, Chiba University, Kamogawa, Chiba 299-5502, Japan; 4 Department of Environmental and Forest Biology, State University of New York College of Environmental Science and Forestry, Syracuse, NY 13210, USA; 5 Current address: Laboratory of Insect Ecology, Graduate School of Agriculture, Kyoto University, Oiwake-cho, Kitashirakawa, Sakyo-ku, Kyoto City, 606-8502, Japan.

## Abstract

Sex is a paradoxical phenomenon because it is less efficient compared with asexual reproduction. To resolve this paradox we need a direct comparison between sexual and asexual forms. In many organisms, however, sexual and asexual forms do not occur in the same habitat, or at the same time. In a few cases where sexual and asexual forms are found in a single population, some (though rare) genetic exchange is usually detected between the two forms. When genetic exchange occurs a direct comparison is impossible. Here we investigate a thrips exhibiting both sexual and asexual forms (lineages) that are morphologically indistinguishable. We examine if the two forms are genetically isolated. Phylogeny based on nuclear genes confirms that the sexual and asexual lineages are genetically differentiated. Thus we demonstrate that the current system has certain advantages over existing and previously used model systems in the evolution of sexual reproduction.

The origin and maintenance of sex is one of the largest puzzles in evolutionary biology because sexual reproduction is accompanied by a number of costs compared with asexual reproduction^1^,^2^,^3^,^4^,^5^,^6^,^7^. Given the significant costs and dominance of sex, the benefit of sexual reproduction must be large to have evolved and be maintained. Although many models have been proposed and tested to explain the superiority of sex over asex^8^,^9^,^10^,^11^,^12^,^13^,^14^, the results are inconclusive. This is partly because of a lack of a comparative analysis of population dynamics between sympatric sexual and asexual populations, although the most popular cost of sex, that is production of males, influences population dynamics. However, such a comparison is almost impossible because for the following reasons. (1) In most organisms, the asexual form is not found or only seen temporarily. (2) In a few species, where the asexual and sexual forms occur at the same time in the same habitat, the asexual lineage is not usually isolated genetically^15^,^16^,^17^,^18^,^19^,^20^,^21^. Therefore, in these species, we cannot directly compare the sexual and asexual populations because those populations are not distinct. For a direct comparison we need an asexual form (lineage) that is genetically isolated from the sympatric sexual counterpart. Such species is an excellent model organism with which to evaluate the relative costs and benefits of sex in an ecological context.

One candidate for a direct comparison is the onion thrips (*Thrips tabaci*) in which sexual and asexual lineages are sympatrically distributed. Although both the sexual and asexual lineages have similar morphologies (Figure 1), previous studies suggested genetic differentiation between the sexual and asexual lineages. Phylogenetic studies using mitochondrial DNA (mtDNA) sequences suggested that the asexual lineage has been derived only once from the sexual lineage and forms a distinct monophyletic group^22^,^23^. The lineage of any individual could be discriminated by its mtDNA sequence using a polymerase chain reaction with sequence specific primers (PCR-SSP)^23^. Under laboratory conditions, the reproductive mode of offspring is always identical with that of the mother^22^,^24^. Thus, all these earlier studies suggested a genetic isolation between the sexual and asexual lineages.

To test the genetic isolation between the lineages in *T. tabaci*, we conducted a molecular phylogenetic analysis of the lineages. We collected adult females from two leek crop fields, discriminated their reproductive mode by the PCR-SSP method and genotyped them at nine microsatellite loci. Using the microsatellite data, we constructed a molecular phylogeny of the lineages.

## Results

**Figure 2:**
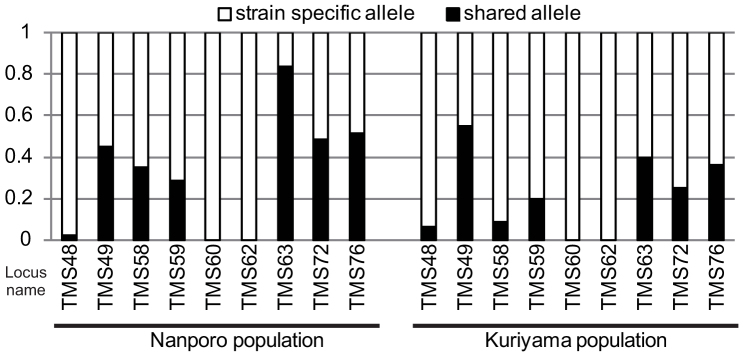
Proportion of lineage-specific alleles at nine microsatellite loci in the two populations. Each bar represents data on each locus from Nanporo (left) and Kuriyama (right) populations. Open bars represent the proportion of lineage-specific alleles, while filled bars represent the proportion of alleles shared between the sexual and asexual lineages.

**Figure 3:**
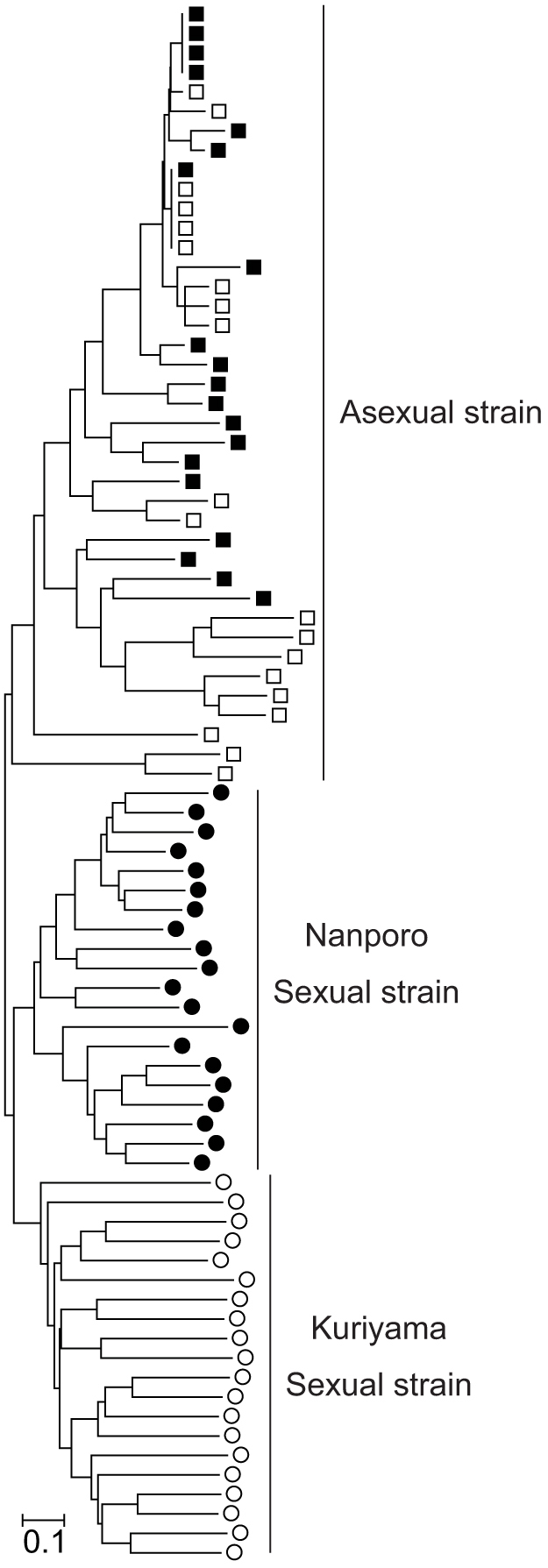
Neighbor-joining tree constructed from pairwise individual genetic distances. Eighty sexual and asexual females from the two sampling locations are represented. Symbols represent the reproductive mode detected by PCR-SSP (square, asexual type; circle, sexual type) and the sampling location (filled, Nanporo; open, Kuriyama) for each individual. The scale bar corresponds to a 10% difference in the genetic distance for a given horizontal branch length.

Using the classification derived from the PCR-SSP method^23^, we discriminated the sexual and asexual females collected in the fields. Among 74 adult females collected at Nanporo, Hokkaido prefecture, Japan, 28 (46) were found to belong to the sexual (asexual) lineage. Similarly, among 74 adult females collected at Kuriyama, Hokkaido prefecture, Japan, 42 (32) were found to belong to the sexual (asexual) lineage. The microsatellite analysis showed that the allele frequencies at all nine loci were significantly different between the sexual and asexual lineages at both locations (Fisher's exact test for all loci with Bonferroni correction; *n* = 40, *p* < 0.0001). Furthermore, lineage-specific alleles were found at all loci (Figures 2). The neighbor-joining tree constructed from the pairwise genetic distances between individuals also revealed that *T. tabaci* could be divided exactly into the two groups: the sexual and asexual females that were based on the classification derived from the PCR-SSP method (Figure 3). Genetic differentiation was observed between the sexual and asexual lineages (*F_ST_* = 0.24330; *p* < 0.0001; permutation tests performed using the computer program Arlequin version 3.5.1.2^25^).

## Discussion

Genetic isolation was observed between the sexual and asexual lineages within the two populations of *T. tabaci* (Figures 2 and 3). This result is consistent with an earlier laboratory study reporting that the offspring produced by each lineage showed the same reproductive mode as their mother^22^,^24^. If genetic exchange between the lineages occurred frequently or the asexual lineage originated repeatedly from the sexual one, we would find several groups including both lineages in the phylogenetic tree. However, the tree showed that each lineage formed a distinct monophyletic group from each other (Figure 3). Thus, our results are consistent with a scenario in which the asexual lineage has a single origin at the sexual lineage, is genetically isolated from the sexual one, and that the genetic diversity within them is acquired through mutations after the establishment of the asexual lineage. Therefore, the sexual and asexual lineages are genetically isolated, assuring a direct comparison between the two reproductive forms.

Because the genetically isolated sexual and asexual lineages of the current thrips are found in the same crop field at the same time (ref. 23, 24 and this study), there should be a mechanism for this coexistence. The competitive exclusion principle of ecology suggests that two or more species sharing the same resources cannot coexist in a community^26^. This might be the reason why we cannot find the coexistence of the sexual and asexual lineages in almost all organisms. Here, we propose a hypothetical mechanism for coexistence in *T. tabaci* that is the density-dependent disturbance by pesticide applications that causes the decrement of resident species, increases available resources in the population, and reduces the interspecific competition for resources. Because *T. tabaci* is an economically important pest insect, a high density of thrips will invoke the pesticide application by farmers. Because the asexual lineage with a high reproductive (growth) rate due to no male production reaches a critical (high) density in a short period, it will suffer from frequent pesticide applications. In contrast, because the sexual lineage with a low reproductive rate takes a long time to reach the critical density, it faces pesticide application at a relatively low frequency. If the high reproductive rate of the asexual lineage is counter-balanced by the frequent pesticide applications, the coexistence will be longstanding. This hypothesis seems to work only in pest insects. Indeed, the assuming factor causing local extinction in the hypothesis is artificial pesticide application. However, under natural conditions where some strong density effects cause local extinction, it could be a general mechanism. Thus, our hypothesis implies that the higher reproductive rate arising from no male production is not always beneficial for asexual individuals. Assuming the pesticide application as the strong density effect, the coexistence of sexual and asexual lineages in *T. tabaci* will have arisen relatively recently. Under this mechanism a higher dispersal rate in the asexual lineage will be selected to avoid the pesticide disturbance than in the sexual lineage. In the phylogeny of individuals, sexual females exhibited a cluster based on the sampling fields, whereas asexual females did not show such a pattern (Figure 3). This pattern difference might have arisen from the difference in dispersal rate between the two lineages. Further investigations focusing on the sympatric distribution of the lineages will provide important insights not only on the species assembly in community ecology but also on the maintenance of sex.

A large number of studies focusing on sex have been performed using intraspecific comparisons between cyclical parthenogenetic (sexual) and obligate parthenogenetic (asexual) lineages or between diploid sexual and polyploid asexual lineages^13^,^27^,^28^,^29^,^30^,^31^,^32^. These sympatric sexual and asexual lineages are useful to test some hypotheses explaining the benefit of sex. However, in these cases, it is almost impossible to test the long-term benefit of sex in the wild because the conditional use of sex by the asexual lineage is known^15^,^16^,^17^,^18^,^19^,^20^,^21^. In contrast, the revealed genetically isolated lineages of *T. tabaci* will be useful to explore the effect of sex in the long-term population dynamics under field conditions. For example, we can expect that the dynamics of genes coding beneficial traits such as pesticide resistance will show a different pattern between the sexual and asexual lineages. In addition, the cost of males in *T. tabaci* is noteworthy because no males were found in the asexual lineage^22^ and the sexual lineage showed female-biased sex ratio^23^,^33^ which gives us the opportunity to test the “cost of males” assumption. Therefore, future investigation using the lineages of *T. tabaci* will provide important insights in the evolution of sex.

## Methods

*T. tabaci* were sampled from a leek crop field in Kuriyama town and Nanporo town, Hokkaido prefecture, Japan on July 30, 2009 and August 10, 2011, respectively. After the sex of each adult was determined under a microscope, genomic DNA was extracted from adult females using a modified Chelex method. Dried individual thrips were crushed in a mixture comprising 5 μl proteinase K (20 mg/ml) and 200 μl 5% Chelex solution (10 mM Tris-HCl; pH 8.0) and incubated at 55°C for more than 12 h in 1.5-ml micro centrifuge tubes. Subsequently, the mixture was boiled at 98°C for 10 min to inactivate proteinase K. Water layer nearby Chelex layer was used as a template DNA.

The reproductive mode of each adult female was determined using a PCR-SSP method^23^. The primer set comprised one primer shared by the lineages (TCOR, 5′-attgcgtaaattattcctaaaagtcca-3′) and two primers (sexual lineage-specific primer TCOS, 5′-aacagcTattctCcttcttttatctC-3′; asexual lineage-specific primer TCOC, 5′-gaacagtatatccacctttatcaacG-3′; the capital letters indicate the lineage-specific nucleotides) that amplified mtDNA of different fragment lengths for each lineage. The composition of the reaction mixture for the PCR-SSP was as follows: 10 μl reaction mixture comprised 5 pmol consensus primer and 2.5 pmol lineage-specific primers, 5 μl 2 × MightyAmp Buffer, 0.1 μl MightyAmp DNA Polymerase (TaKaRa, Otsu, Japan; 1.25 U/μl), and 0.5 μl template DNA. PCR-SSP was performed using a 2720 Thermal Cycler (Applied Biosystems, Foster City, CA) by the following temperature cycles: initial denaturation for 2 min at 98°C, followed by 35 cycles of denaturation and annealing of 10 sec at 98°C and 1 min at 60°C, respectively, and the final extension for 1 min at 68°C. The fragment length of PCR products was detected by 1% agarose gel electrophoresis using 5 μl of 100 bp DNA Ladder (TaKaRa, Otsu, Japan) and ethidium bromide staining.

After identification of the lineage, 20 randomly chosen adult females from each lineage were genotyped at nine microsatellite loci. To amplify these microsatellite loci, a 2720 Thermal Cycler (Applied Biosystems, Foster City, CA) was used for PCR that was performed as follows: initial denaturation of 2 min at 96°C followed by 35 cycles of denaturation for 5 sec at 98°C, annealing for 30 sec at temperature specific for each primer pair, extension for 1 min at 68°C, and final extension of 1 min at 68°C. The primer sequences and annealing temperature for these microsatellite loci were described previously^34^. Each 10 μl reaction mixture comprised 2 pmol primer, 5 μl 2 × MightyAmp Buffer, 0.1 μl MightyAmp DNA Polymerase (TaKaRa, Otsu, Japan; 1.25 U/μl), and 1 μl template DNA. One-μl PCR product was electrophoresed with 0.5 μl size standard 400 (Beckman-Coulter, Fullerton, CA) on a CEQ-8000 Genetic Analyzer (Beckman-Coulter, Fullerton, CA). Data of allele frequencies were analyzed using the program Arlequin version 3.5.1.2^25^.

The microsatellite analysis data were used to infer phylogenetic relationships between the females of both lineages. As a measure of pairwise genetic differences among females, we used the following allele-sharing distance^35^,^36^: 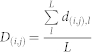
where *L* is the total number of loci and *d_(i, j),l_* = 0, if the individuals *i* and *j* have identical genotypes at the locus *l*, *d_(i, j),l_* = 0.5 (if they share a single allele), and *d_(i, j),l_* = 1.0, if they have no common allele. A neighbor-joining tree was constructed from the pairwise distance matrix using the software MEGA version 5^37^.

## Author Contributions

K.K. and E.H. conceptualized, planned and coordinated the study; K.K. collected and analysed the data; all authors discussed the results; K.K. and J.Y. wrote the paper.

## Figures and Tables

**Figure 1:**
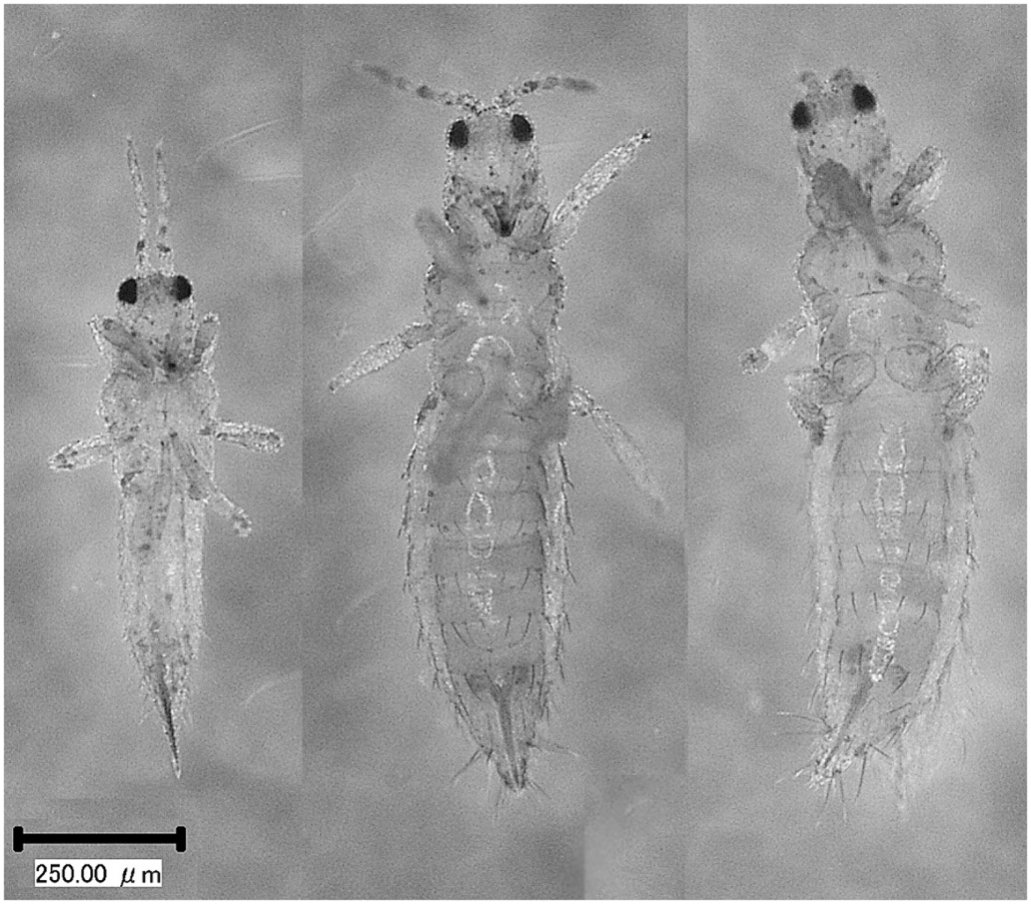
A sexual male (left), sexual female (center), and an asexual female (right) of the onion thrips (*Thrips tabaci*).
